# Metabolic Hijacking of Hexose Metabolism to Ascorbate Synthesis Is the Unifying Biochemical Basis of Murine Liver Fibrosis

**DOI:** 10.3390/cells12030485

**Published:** 2023-02-02

**Authors:** Diren Beyoğlu, Pinzhu Huang, Disha Skelton-Badlani, Christine Zong, Yury V. Popov, Jeffrey R. Idle

**Affiliations:** 1 Department of Pharmaceutical and Administrative Sciences, College of Pharmacy and Health Sciences, Western New England University, Springfield, MA 01119, USA; 2 Arthur G. Zupko Institute for Systems Pharmacology and Pharmacogenomics, Arnold & Marie Schwartz College of Pharmacy and Health Sciences, Long Island University, Brooklyn, NY 11201, USA; 3 Division of Gastroenterology, Hepatology and Nutrition, Beth Israel Deaconess Medical Center, Harvard Medical School, 330 Brookline Avenue, Boston, MA 02215, USA; 4 Department of BioMedical Research, University of Bern, 3008 Bern, Switzerland

**Keywords:** fibrosis, mouse model, metabolomics, liver, glucose, galactose, ascorbate, collagen, metabolic reprogramming, aldose reductase

## Abstract

We wished to understand the metabolic reprogramming underlying liver fibrosis progression in mice. Administration to male C57BL/6J mice of the hepatotoxins carbon tetrachloride (CCl4), thioacetamide (TAA), or a 60% high-fat diet, choline-deficient, amino-acid-defined diet (HF-CDAA) was conducted using standard protocols. Livers collected at different times were analyzed by gas chromatography–mass spectrometry-based metabolomics. RNA was extracted from liver and assayed by qRT-PCR for mRNA expression of 11 genes potentially involved in the synthesis of ascorbic acid from hexoses, *Gck*, *Adpgk*, *Hk1*, *Hk2*, *Ugp2*, *Ugdh*, *Ugt1a1*, *Akr1a4*, *Akr1b3*, *Rgn* and *Gulo*. All hepatotoxins resulted in similar metabolic changes during active fibrogenesis, despite different etiology and resultant scarring pattern. Diminished hepatic glucose, galactose, fructose, pentose phosphate pathway intermediates, glucuronic acid and long-chain fatty acids were compensated by elevated ascorbate and the product of collagen prolyl 4-hydroxylase, succinate and its downstream metabolites fumarate and malate. Recovery from the HF-CDAA diet challenge (F2 stage fibrosis) after switching to normal chow was accompanied by increased glucose, galactose, fructose, ribulose 5-phosphate, glucuronic acid, the ascorbate metabolite threonate and diminished ascorbate. During the administration of CCl4, TAA and HF-CDAA, aldose reductase *Akr1b3* transcription was induced six- to eightfold, indicating increased conversion of glucuronic acid to gulonic acid, a precursor of ascorbate synthesis. Triggering hepatic fibrosis by three independent mechanisms led to the hijacking of glucose and galactose metabolism towards ascorbate synthesis, to satisfy the increased demand for ascorbate as a cofactor for prolyl 4-hydroxylase for mature collagen production. This metabolic reprogramming and causal gene expression changes were reversible. The increased flux in this pathway was mediated predominantly by increased transcription of aldose reductase *Akr1b3*.

## 1. Introduction

The excessive accumulation in the liver of extracellular matrix proteins, particularly collagen, is the hallmark of liver fibrosis and is a wound-healing response to liver injury. Liver fibrosis in developed nations is caused mainly by alcohol abuse, chronic HCV infection and rising prevalence of nonalcoholic fatty liver disease (NAFLD) and nonalcoholic steatohepatitis (NASH) due to obesity epidemics [1,2]. Liver fibrosis develops in distinct histological patterns (post-necrotic, metabolic, and biliary) but is present in the majority of chronic liver diseases regardless of etiology, and ultimately progresses to irreversible cirrhosis and to hepatocellular carcinoma (HCC) [2,3]. Chronic liver diseases involving fibrosis have resulted in significant and increasing global morbidity and mortality [4]. The principal treatment option for end-stage cirrhosis is transplantation. Only about one-quarter of those on the U.S. liver transplantation waiting list in 2020 received a transplant [5], thereby underlining the need for novel therapies to halt or reverse fibrosis. There is still a lack of an effective treatment for liver fibrosis [6], and the detailed mechanism by which it occurs or may be reversed is incompletely understood [2,4]. In response to hepatocyte insult, the role of hepatic stellate cell (HSC) differentiation to collagen-producing myofibroblasts is well known [2]. The generation of fibrogenic cytokines such as TGF-β and PDGF by macrophages and lymphocytes in response to hepatocyte injury has been studied extensively [2]. The role of natural killer (NK) cells in destroying activated HSCs as a component of hepatic innate immunity has also been widely investigated [3]. More recently, omics methodologies are said to have revealed mechanistic insights into the biology of liver fibrosis [6]. Specifically, new mechanisms of fibrogenic crosstalk within the injured liver have emerged from cell-specific mapping of fibrosis-activated transcriptional networks, in particular in the transition from NAFLD to NASH [7]. The omics reports on the mechanisms of liver fibrosis to date have focused on genomics and transcriptomics [6,7]. Earlier research into the mechanisms of chemical insult of the liver was through the study of their biochemical basis. For example, the investigation of carbon tetrachloride hepatotoxicity centered on the examination of microsomal lipid peroxidation [8] and galactosamine-induced liver cell necrosis focused on intracellular Ca^2+^ accumulation [9]. In the intervening 50 years, the landscape of medical research has altered markedly. Contemporary molecular and cell biology offer a toolbox with which we can define the complexities of cellular processes at a previously unimagined level of detail. That notwithstanding, it is the underlying metabolic activities that feed the cellular and molecular processes that are often understudied. To comprehend fully and to treat and prevent liver fibrosis, it would be advantageous to understand first the role of metabolism in its causation. The biochemical basis of disease can readily be probed using another omics technology, that of mass-spectrometry-based metabolomics [10,11,12]. Metabolomics can be employed to help illuminate the earliest stages of the fibrotic response to liver injury—the metabolic processes involved in the hepatic synthesis of mature collagen fibers. It is well established that prolyl 4-hydroxylase catalyzes the formation of 4-hydroxyproline in collagens and related proteins that have collagen-like amino acid sequences. Hydroxylation of proline residues occurs at -X-Pro-Gly- sequences and resultant 4-hydroxyproline residues stabilize the collagen triple helix under physiological conditions. In contrast, collagen chains that contain no 4-hydroxyproline cannot fold into triple helices that are stable at body temperature. This hydroxylation requires Fe^2+^, O_2_, ascorbate and 2-oxoglutarate, the latter undergoing oxidative decarboxylation to succinate [13]. Murine models are employed to understand hepatic stellate cell activation and mechanisms of fibrosis [14]. In principle, such animal models may also be used to define the metabolic pathways at the core of the fibrogenic mechanisms by the use of mass spectrometry-based metabolomics.

Here, we have utilized three distinct methodologies for the generation of liver fibrosis and cirrhosis in the mouse, carbon tetrachloride (CCl4) and thioacetamide (TAA) administration [15] as models representing post-necrotic type fibrosis, together with a high-fat, choline-deficient, amino-acid-defined (HF-CDAA) diet as a model of metabolic-type fibrosis characteristic of NAFLD/NASH [16]. Although both CCl4 and TAA result in selective hepatocyte death, which triggers inflammatory and progressive fibrotic response, the mechanisms of CCl4 and TAA-induced fibrogenesis and underlying metabolic changes have not been fully elucidated [15]. In the case of HF-CDAA mice, steatohepatitis with dietary fat-driven dysregulation of lipid metabolism genes has been reported, followed by rapidly progressing fibrosis leading to cirrhosis, portal hypertension and HCC by 24 week [16]. As with CCl4 and TAA, the biochemical basis of HF-CDAA-induced fibrosis and underlying metabolic reprogramming is also uncertain. Here, we report that all three hepatic insults leading to fibrosis in mouse liver share a canonical pathway that appropriates the glucose metabolic pathways, including glycolysis and the pentose phosphate pathway. The resultant enhanced glucose flux to ascorbate is necessary to deliver proline 4-hydroxylation for the synthesis and stabilization of mature collagen, which we propose as the underlying biochemical basis of murine liver fibrosis.

## 2. Materials and Methods

### 2.1. Animals

Male C57BL/6J inbred mice were purchased from Jackson Laboratories (strain #000664, Bar Harbor, ME, USA). All mice were acclimatized for 1 week before experiments and housed in a 12:12 h dark–light cycle and fed a standard rodent chow and tap water ad libitum. Animal experiments were approved by the Institutional Animal Care and use Committee (Beth Israel Deaconess Medical Center (BIDMC); protocols158-2008, 004-2012 and 003-2021) and were conducted in the same facility room at BIDMC.

#### 2.1.1. Fibrosis Model #1. Carbon Tetrachloride-Induced Pan-Lobular Liver Fibrosis

Progressive CCl4-induced fibrosis was induced according to an optimized escalating-dose protocol, as described in detail previously [17]. Briefly, male 7–8-week-old C57BL/6J mice (*n* = 4; controls, *n* = 4) were treated with escalating doses of CCl4 (50/50 vol. with mineral oil) three times per week by oral gavage for six weeks, starting with 0.875 mL/kg (1st dose, week 1), 1.75 mL/kg (week 1–4) and 2.5 mL/kg (dose, week 4–6) [15,17]. Animals were euthanized 3 days after the last dose of CCl4.

#### 2.1.2. Fibrosis Model #2. Thioacetamide-Induced Pan-Lobular Liver Fibrosis

Progressive TAA-induced fibrosis was induced according to an optimized escalating-dose protocol, as described in detail previously [17]. Male 7–8-week-old C57BL/6J mice (*n* = 11; controls, *n* = 4) were treated with escalating doses of TAA dissolved in 200 μL of PBS, intraperitoneally three times a week, starting with 50 mg/kg (week 1), 100 mg/kg (week 1–2), 200 mg/kg (week 2–4), 300 mg/kg (week 4–5), and 400 mg/kg (week 6) [15,17]. Animals were euthanized 3 days after the last dose of TAA.

#### 2.1.3. Fibrosis Model #3. Choline-Deficient, Amino-Acid-Defined Diet with High-Fat Content

The choline-deficient, amino acid-defined diet (CDAA) containing 60% fat by calories (HF-CDAA) was administered to 8-wk-old male C57BL/6J mice (*n* = 38; controls, *n* = 4) as described in. detail previously [16]. The diet was obtained from Research Diets Inc. (New Brunswick, NJ, USA). Regular chow (Purina 5008) was used as normal diet control [16]. Animals were euthanized at week 4 (*n* = 5), 8 (*n* = 8 plus *n* = 12 retained for the spontaneous recovery experiment; see below), 12 (*n* = 4) and 24 week (*n* = 9) of HF-CDAA feeding, representing fibrosis stages F1, F2, F3 and F4, respectively [16].

#### 2.1.4. Fibrosis Model #4. Spontaneous Recovery from HF-CDAA-Induced Liver Fibrosis

The reversibility of effects on the liver metabolome during spontaneous regression of fibrosis was investigated in mice treated with the HF-CDAA diet for 8 week to establish advanced fibrosis (F2), then switched to regular chow and allowed to recover spontaneously. Animals were euthanized at previously established 1 (*n* = 5), 4 (*n* = 3) and 12 week (*n* = 4) time-points of recovery for sampling [16].

In all experiments, euthanasia was conducted by exsanguination with cardiac puncture under general anesthesia and the livers promptly excised, weighed and snap-frozen in liquid nitrogen. For all investigations, liver specimens were stored at −80 °C prior to shipment to LIU-Brooklyn for mass spectrometry metabolome analysis.

### 2.2. Chemicals and Solvents

4-Chlorophenylacetic acid, ultrapure pyridine and BSTFA/TMCS were obtained from MilliporeSigma (Burlington, MA, USA). LC-MS grade methanol and acetonitrile were purchased from Thermo Fisher Scientific (Waltham, MA, USA).

### 2.3. Analysis of Mouse Livers by Gas Chromatography-Mass Spectrometry (GC-MS)

Frozen mouse livers (*n* = 65; see above) were transported on dry-ice from BIDMC to LIU Brooklyn by courier. On receipt, samples were stored at −80 °C. For preparation of liver extracts for GC-MS analysis, approximately 50 mg of each mouse liver (weighed accurately) was homogenized in aqueous methanol (50:50 *v*/*v*; 1.0 mL) containing 4-chlorophenylacetic acid (4-CPAA; 0.1 mM) as internal standard for GC-MS. All samples were kept on ice and homogenized for 20 sec with a Fisherbrand bead homogenizer, spun at 14,500 g for 20 min at 4 °C. Supernatants were split into 2 × 400 μL aliquots to each of which was added acetonitrile (400 μL), vortexed and stood on ice for 20 min and then spun at 14,500 g for 20 min at 4 °C. Supernatants (700 μL each) were kept at −20 °C until analysis.

For GC-MS analysis, to each supernatant transferred in a screw-top glass tube was added acetonitrile (1 mL) and reduced to dryness in a Savant evaporating centrifuge in vacuo at 40 °C for 2 h. To each dry residue was added ultra-pure pyridine (100 μL) and BSTFA containing 1% TMCS (100 μL), the tubes tightly capped, vortexed and heated at 75 °C for 30 min to silylate amino acids, organic acids and sugars to render them volatile for analysis by GC-MS [18,19].

Samples were analyzed by GC-MS using slight variations of our published methods [18,19,20]. QC samples, prepared from aliquots of each sample, were included and analyzed as the first five samples injected and then interspersed between every tenth analytical sample. Samples (1.0 µL) were injected in duplicate using an Agilent 7683B liquid sampler into an Agilent 6890N gas chromatograph with an Agilent 5975B mass selective detector operating under electron impact ionization at 70 eV. The front inlet was operated in splitless mode at 280 °C and an HP5-MS column (60 m; i.d. 250 µm; film thickness 0.25 µm) subjected to a temperature program of 70 °C for 3 min, 10 deg/min to 250 °C, 10 deg/min to 300 °C, held for 8 min (run time 34 min). Mass spectra were collected from *m/z* 35.0 to 650.0. In each 34 min chromatogram, in addition to background peaks and the internal standard, 68 peaks deriving from 58 discrete metabolites were identified by comparison of their mass spectra with the NIST 14 Library (MS Wil GmbH, Wil, Switzerland) that contained 276,248 mass spectra from 242,466 compounds and by comparison of their retention times with an in-house collection of 120 authentic standards. Each annotated peak was quantitated as a peak area ratio (PAR) from its peak area/internal standard peak area using AutoQuant in the on-board Agilent ChemStation software [21,22,23,24,25,26].

### 2.4. Quantitative RT-PCR

**Figure 1:**
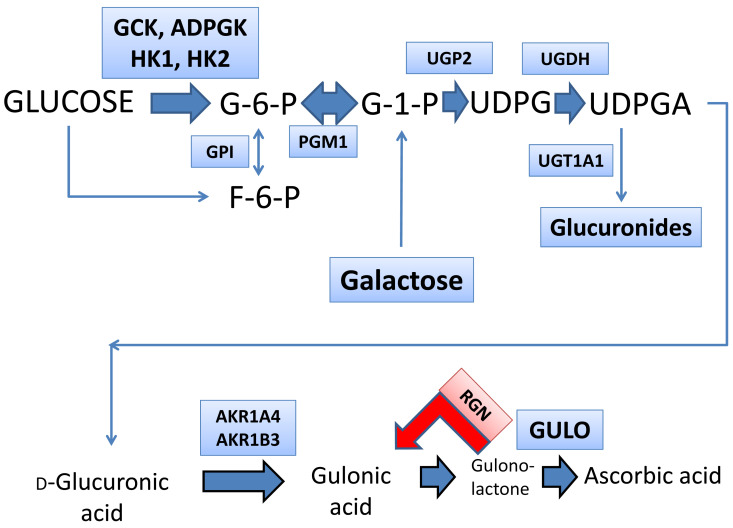
The de novo synthesis of ascorbic acid showing potential enzymes involved whose mRNA expression was determined using quantitative RT-PCR. Intermediates are: G-6-P, glucose 6-phosphate; F-6-P, fructose 6-phosphate; G-1-P, glucose 1-phosphate; UDPG, uridine diphosphate glucose; UDPGA, uridine diphosphate glucuronic acid. The enzymes are: GCK, glucokinase (HK4; EC 2.7.1.1); ADPGK, ADP-dependent glucokinase (EC 2.7.1.147); HK1, hexokinase 1 (EC 2.7.1.1); HK2, hexokinase 2 (EC 2.7.1.1); GPI, glucose 6-phosphate isomerase (EC 5.3.1.9); PGM1, phosphoglucomutase 1 (EC 5.4.2.2); UGP2, UDP-glucose pyrophosphorylase 2 (EC 2.7.7.9); UGDH, UDP glucose 6-dehydrogenase (EC 1.1.1.22); UGT1A1, UDP glucuronosyltransferase family 1 member A1 (EC 2.4.1.17); AKR1A4, aldo-keto reductase family 1, member A1 (aldehyde reductase; EC 1.1.1.2); AKR1B3, aldo-keto reductase family 1, member B3 (aldose reductase; EC 1.1.1.21); RGN, regucalcin (gluconolactonase; EC 3.1.1.17); and GULO, gulonolactone oxidase (EC 1.1.3.8).

A sample (300–400 mg) of liver tissue (*n* = 65; see above) from two lobes was homogenized, and total RNA was extracted using TRIzol (Invitrogen), and 1ug of total RNA was reverse transcribed as described previously [27]. Relative transcript levels were quantified with real-time RT-PCR on a LightCycler 1.5 instrument (Roche, Mannheim, Germany) using the TaqMan methodology and second derivative analysis option as described previously [27]. TaqMan probes (dual-labeled with 5-FAM and 3-TAMRA) and primers (Supplemental Table S1) were designed using the Primer Express software (Perkin Elmer, Wellesley, MA, USA), synthesized at Eurofins Scientific, or obtained commercially from Thermo Fisher Scientific. The housekeeping gene beta-2-microglobulin (b2MG) was amplified in parallel reactions for normalization. Gene expression was determined in relation to the de novo synthesis of ascorbic acid from glucose, as shown in Figure 1. The pathway from glucose and galactose to ascorbic acid can be divided into three phases: hexose phosphorylation (genes investigated: *Gck*, *Adpgk*, *Hk1*, and *Hk2*); the synthesis of glucuronic acid from glucose 1-phosphate (genes investigated: *Ugp2*, *Ugdh*, and *Ugt1a1*); and the synthesis of ascorbic acid from glucuronic acid (genes investigated: *Akr1a4*, *Akr1b3*, *Rgn*, and *Gulo*). Details of the assays and primers are given in Supplemental Table S1.

### 2.5. Hepatic 4-Hydroxyproline Determination

Hepatic collagen content was determined as relative 4-hydroxyproline (µg/g liver) in 100 to 200 mg liver samples from two different lobes (representing 10% of whole liver) after hydrolysis in 6 N HCl for 16 h at 110 °C, as described [16,28].

### 2.6. Statistical Methods

Nonparametric statistics were employed throughout using GraphPad Prism 9.5.0 (GraphPad Software, San Diego, CA, USA). For comparisons between 2 groups, the Mann–Whitney test was used. For comparisons between 3 or more groups, the Kruskal–Wallis test was used. In Figures and Tables, results are expressed as mean ± s.e.m.

## 3. Results

### 3.1. Metabolomics

#### 3.1.1. Histology and Biochemistry of the Livers

**Figure 2:**
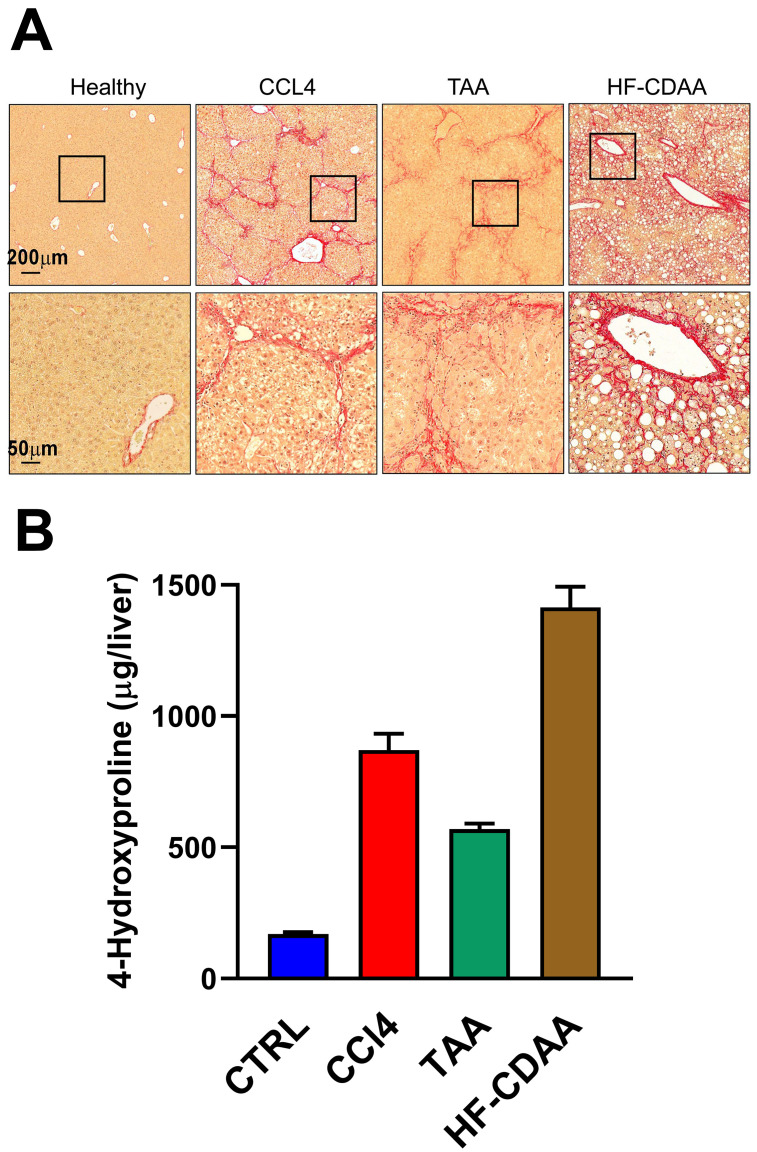
(**A**). Histologically distinct hepatic fibrosis patterns in three utilized chronic liver injury models. Liver sections were prepared from healthy animals, mice subjected to repeated administration carbon tetrachloride (CCl4), thioacetamide (TAA) and feeding with choline-deficient, amino-acid-defined, 60% high-fat diet (HF-CDAA), respectively. Representative low- (top, ×50) and high (bottom, ×200)-magnification images of collagen staining (Sirius red) demonstrated robust “post-necrotic type” bridging fibrosis in livers with CCL4 and TAA-induced models. Advanced “chicken wire” perisinusoidal fibrosis characteristic of “metabolic-type” fibrosis observed in NAFLD/NASH develops in livers of HF-CDAA-fed mice. Healthy liver from untreated strain-, sex-, and age-matched mice used as control for comparison. Scale bar: 200 μm (top), 50 μm (bottom). (**B**). 4-Hydroxyproline levels (µg/liver) for control-diet-fed (CTRL; 6 h), CCl4-treated (CCl4; 6 h), TAA-treated (TAA; 6 h), and HF-CDAA-diet-fed (HF-CDAA; 8 h). Differences between CTRL and each treatment group *p* < 0.0001 (Mann–Whitney test).

Three mechanistically distinct mouse models of liver fibrosis were studied. Figure 2A shows the advanced and histologically distinct fibrosis pattern of each model: TAA or CCL4 resulted in bridging, post-necrotic-type scarring with fibrotic septa resembling that of chronic viral hepatitis B and C, whereas HF-CDAA feeding led to perisinusoidal, “chicken wire” fibrosis characteristic of metabolic-type scarring in NAFLD/NASH and alcohol-related liver disease. Figure 2B shows hepatic 4-hydroxyproline levels (µg/liver) that were highly statistically significantly elevated above control-diet-fed animals for each fibrogenic treatment, 5.4-fold for CCl4 treatment, 3.4-fold for TAA treatment, and 8.4-fold for the HF-CDAA-diet-fed animals (*p* < 0.0001).

#### 3.1.2. Fibrosis Model #1. CCl4-Induced Pan-Lobular Liver Fibrosis

**Figure 3:**
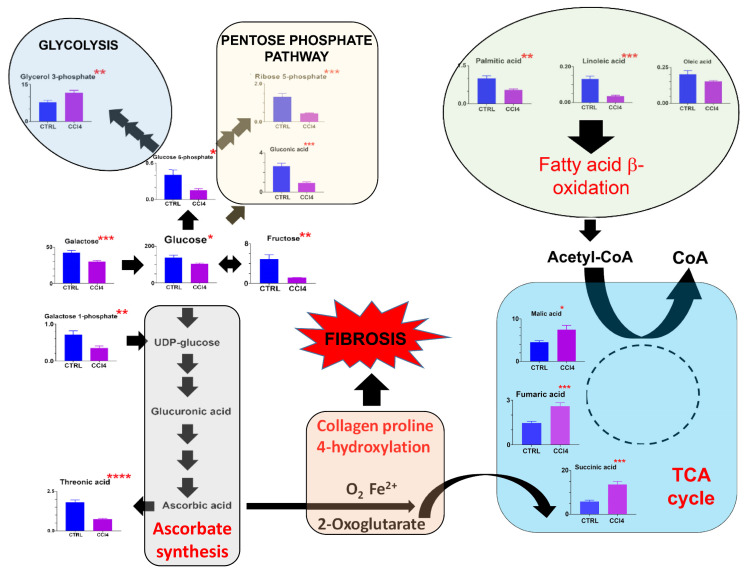
The appropriation of glucose metabolism for ascorbate synthesis in the livers of mice administered carbon tetrachloride * = *p* < 0.05; ** = *p* < 0.01; *** = *p* < 0.001; **** = *p* < 0.0001.

The chronic administration of CCl4 had a profound effect on the hepatic metabolome. First, there were statistically significant diminutions in hepatic glucose and its metabolites glucose 6-phosphate and fructose, together with the pentose phosphate pathway metabolites gluconic acid and ribose 5-phosphate (Table 1; Figure 3). In addition, galactose, galactose 1-phosphate and galacturonic acid were all reduced in the liver. Furthermore, maltose, a disaccharide comprising two glucose molecules, which can be converted to glucose by α-1,4-glucosidase [29], was diminished 6-fold in the liver. As can be seen in Figure 3, there wsa a global diminution in glucose- and galactose-related metabolites and presumably, therefore, a shortfall in glycolysis to feed the TCA cycle. However, it would appear that CCl4 treatment promoted long-chain fatty acid β-oxidation in order to compensate for glycolysis, as judged by the diminished hepatic concentration of the fatty acids palmitic, linoleic, and oleic acids. As a result, the TCA metabolites succinate, fumarate, and malate were all significantly elevated. It should be noted that threonic acid, a terminal metabolite and oxidation product of ascorbate [30], was diminished by CCl4 treatment presumably as a result of the utilization of ascorbate in the 4-hydroxylation of proline [13]. Unfortunately, in this experiment, we did not obtain a direct measurement of ascorbate concentration.

#### 3.1.3. Fibrosis Model #2. TAA-Induced Pan-Lobular Liver Fibrosis

**Figure 4:**
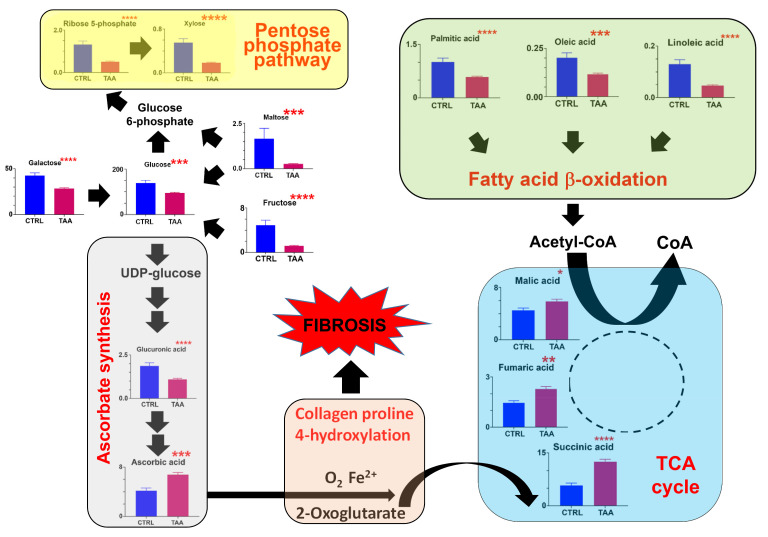
The appropriation of glucose metabolism for ascorbate synthesis in the livers of mice administered thioacetamide * = *p* < 0.05; ** = *p* < 0.01; *** = *p* < 0.001; **** = *p* < 0.0001.

The administration of TAA also had a profound effect on the hepatic metabolome, with a highly statistically significant decrease in glucose, fructose, glucuronic acid, galactose, galacturonic acid, maltose, and the pentose phosphate metabolites ribose 5-phosphate and xylose (Table 2; Figure 4). Furthermore, a 60% increase in hepatic ascorbate was observed but its metabolite threonic acid was undetected. As Figure 4 shows, there was a clear flux of glucose, galactose, and their precursors and metabolites towards ascorbate, which is consistent with the production of mature collagen. Increased proline 4-hydroxylation uses 2-oxoglutarate, which is converted into succinate and onwards to fumarate and malate in the TCA cycle (Figure 4). Interestingly, the oncometabolite 2-hydroxyglutarate [24,31,32] was highly significantly elevated fourfold in the liver of TAA-treated mice (Table 2) as it was in CCl4-treated mice (Table 1). Fatty acid β-oxidation again appeared to be increased as evidenced by the diminution of hepatic fatty acids as a compensation for a shortfall in glycolysis and, therefore, cellular energy (Figure 4).

#### 3.1.4. Fibrosis Model #3. HF-CDAA Diet-Induced Metabolic-Type Liver Fibrosis

**Figure 5:**
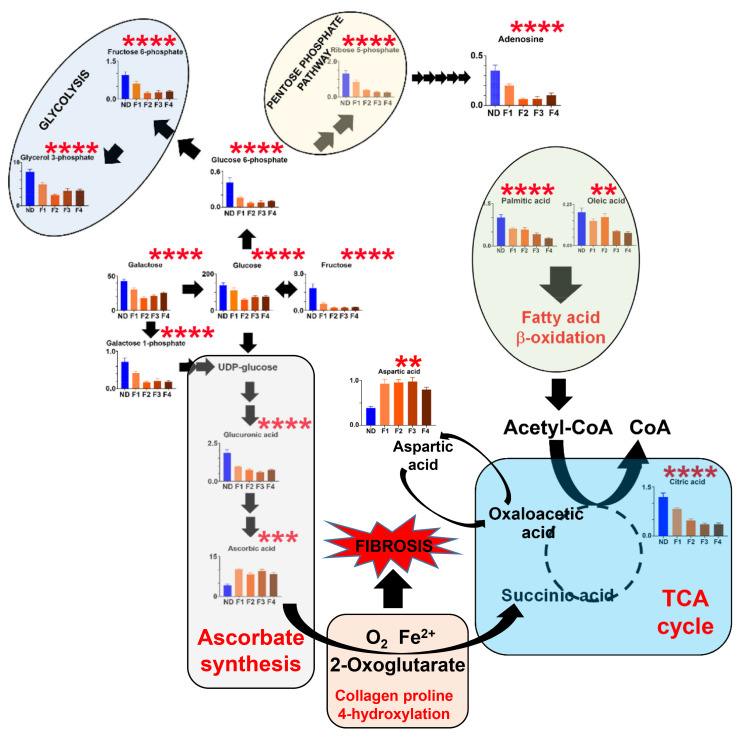
The appropriation of glucose metabolism for ascorbate synthesis in the livers of mice administered choline-deficient, amino-acid-defined diets with high fat content ** = *p* < 0.01; *** = *p* < 0.001; **** = *p* < 0.0001.

This dietary model of steatohepatitis provided the most detailed information regarding the hijacking of glucose and galactose metabolism for the purposes of increased ascorbate synthesis required for de novo fibrogenesis. As depicted in Figure 5, glucose, galactose, fructose, glucose 6-phosphate and galactose 1-phosphate all significantly declined (Kruskal–Wallis ANOVA; *p* < 0.0001) from the healthy, normal chow diet levels through 4, 8, 12 and 24 week on the HF-CDAA diet [16]. Additionally, the glycolytic metabolites fructose 6-phosphate and glycerol 3-phosphate, together with the pentose phosphate pathway metabolite ribose 5-phosphate, similarly significantly declined (*p* < 0.0001). In the ascorbate synthesis pathway, glucuronic acid was similarly significantly depleted (*p* < 0.0001) leading to a significant surge in ascorbate synthesis (*p* < 0.001), which commenced as early as the F1 stage of fibrosis (Figure 5; Table 3). Furthermore, fatty acid β-oxidation appeared to increase with fibrosis progression with a decline in hepatic palmitic acid (*p* < 0.0001) and oleic acid (*p* < 0.01) levels. Succinate was not detected in this experiment and, therefore, the anaplerotic contribution of proline 4-hydroxylation to the TCA cycle could not be directly evaluated. However, the significant decline (*p* < 0.0001) in citrate concentration during fibrosis progression suggested that fatty acid β-oxidation was insufficient to replenish the TCA cycle. Nevertheless, the significant increase in aspartate (*p* < 0.01) during fibrosis progression may indicate elevated cataplerosis from oxaloacetate by aspartate aminotransferase (Figure 5). Elevated oxaloacetate may be formed in the TCA cycle via fumarate and malate from succinate delivered by the decarboxylation of 2-oxoglutarate by proline 4-hydroxylase [33]. Finally, adenosine concentrations in the liver dropped significantly (*p* < 0.0001) over the progression of fibrosis. Adenosine is derived in multiple steps from ribose 5-phosphate [34], which was also diminished during fibrosis progression, in the pentose phosphate pathway (Figure 5). However, in all probability, the determination of adenosine by GC-MS may reflect a greater pool of adenosine phosphates, since ATP is known to have a turnover time of around 1 sec [35] and the resulting AMP and ADP are likely dephosphorylated to adenosine during the GC-MS analysis [36].

#### 3.1.5. Fibrosis Model #4. Spontaneous Reversal of HF-CDAA-Diet-Induced Fibrosis

**Figure 6:**
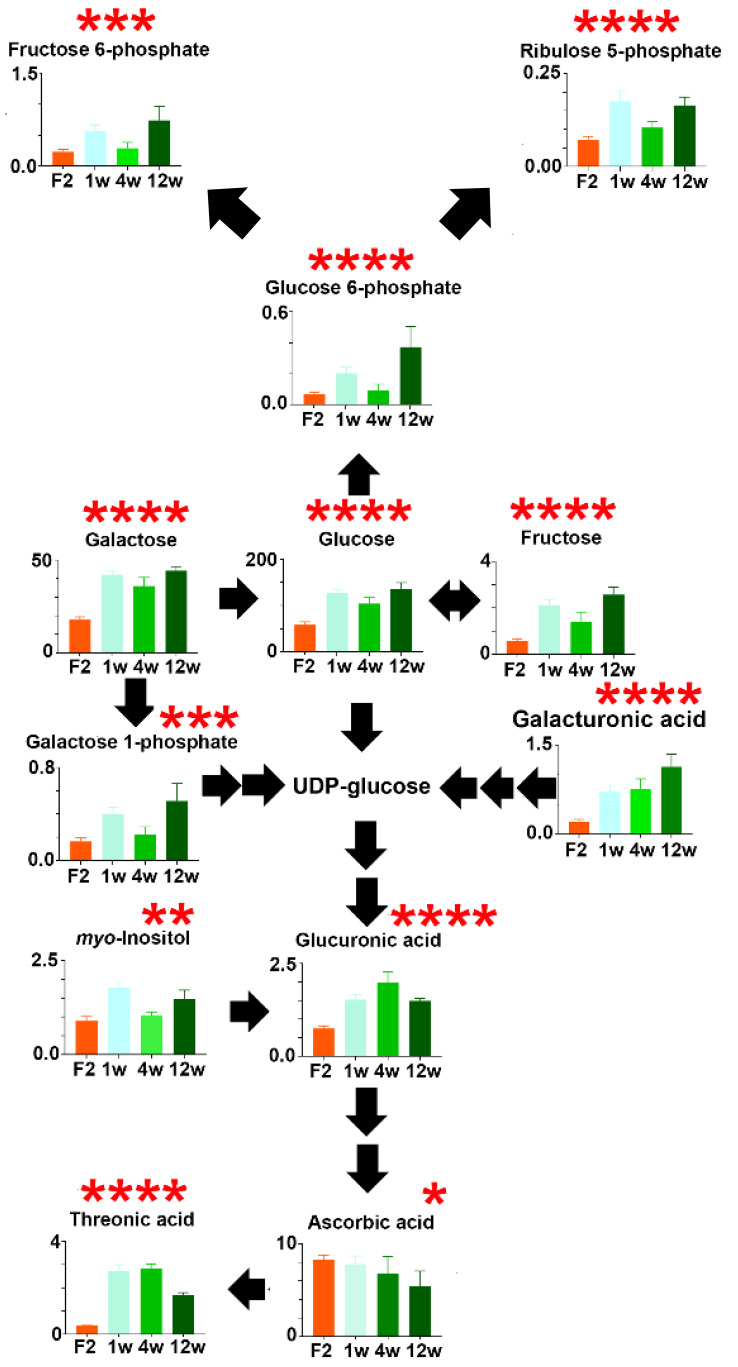
Changes in glucose, galactose and ascorbate metabolism during spontaneous recovery of HF-CDAA-induced liver fibrosis (F2) over 1–12 week * = *p* < 0.05; ** = *p* < 0.01; *** = *p* < 0.001; **** = *p* < 0.0001.

Switching the HF-CDAA fibrogenic diet to regular chow at 8 week of treatment and at stage F2 had a dramatic effect on the liver metabolome over the next 12 week (Figure 6). As early as 1 week after cessation of the fibrogenic diet, hepatic levels of glucose, galactose and fructose rose significantly (*p* < 0.0001) by more than twofold. The glycolysis metabolite fructose 6-phosphate (*p* < 0.001) and the pentose phosphate pathway metabolite ribulose 5-phosphate (*p* < 0.0001) both rose significantly during the recovery phase. Galactose 1-phosphate, galacturonic acid, glucuronic acid, *myo*-inositol and glucuronic acid all rose significantly during 12 week of spontaneous recovery. Interestingly, ascorbate liver levels fell 50% (*p* < 0.05) during the recovery phase. In stark contrast, the ascorbate terminal metabolite threonic acid rose two- to threefold (*p* < 0.0001), especially during the first 4 week of recovery (Figure 6). These findings bolster the interpretation that glucose and galactose metabolism is diverted to ascorbate production to help drive fibrosis, which is reversed once the fibrotic driving force of the HF-CDAA diet is removed.

### 3.2. Changes in Relevant Metabolism Gene Expression by qRT-PCR after Chronic Administration of CCl4 and TAA

**Figure 7:**
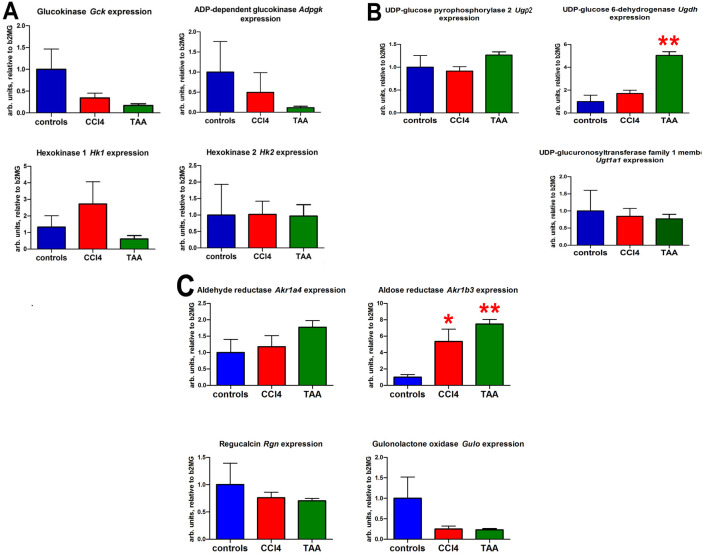
Hepatic expression of mouse genes involved in ascorbic acid synthesis from glucose and galactose after administration of CCl4 or TAA. (**A**). The phosphorylation of hexoses pathway. (**B**). The synthesis of glucuronic acid from glucose 1-phosphate pathway. (**C**). The synthesis of ascorbic acid from glucuronic acid pathway * = *p* < 0.05 ** = *p* < 0.01 compared to controls.

Figure 7A shows the effect of CCl4 treatment on hepatic gene expression for four genes potentially involved in phosphorylation of glucose and galactose, *Gck*, *Adpgk*, *Hk1* and *Hk2*. No statistically significant changes were observed for any of these genes after CCl4 administration relative to control animals. However, expression of *Gck* and *Adpgk* was reduced 7-fold. In Figure 7B, none of the three genes involved in the synthesis of glucuronic acid from glucose 1-phosphate (*Ugp2*, *Ugdh* and *Ugt1a1*) showed any statistically significant alterations in the liver after administration of CCl4 compared to control mice maintained on a normal diet alone. Figure 7C displays hepatic qRT-PCR findings for four genes involved in the conversion of glucuronic acid to ascorbic acid. Expression of the aldose reductase (*Akr1b3*) gene was altered (5.7-fold median increase; *p* = 0.03) by the administration of CCl4.

Hepatic gene expression changes after administration of TAA compared to control mice are displayed in Figure 7A–C. The expression of both *Ugdh* and *Akr1b3* was statistically significantly elevated (*p* = 0.005) after TAA dosing, indicating increased flux from glucose 1-phosphate to glucuronic acid and on to ascorbic acid (Figure 1).

### 3.3. Hepatic Gene Expression Changes by qRT-PCR during Choline-Deficient, Amino-Acid-Defined, 60% High-Fat Diet (HF-CDAA) Feeding

**Figure 8:**
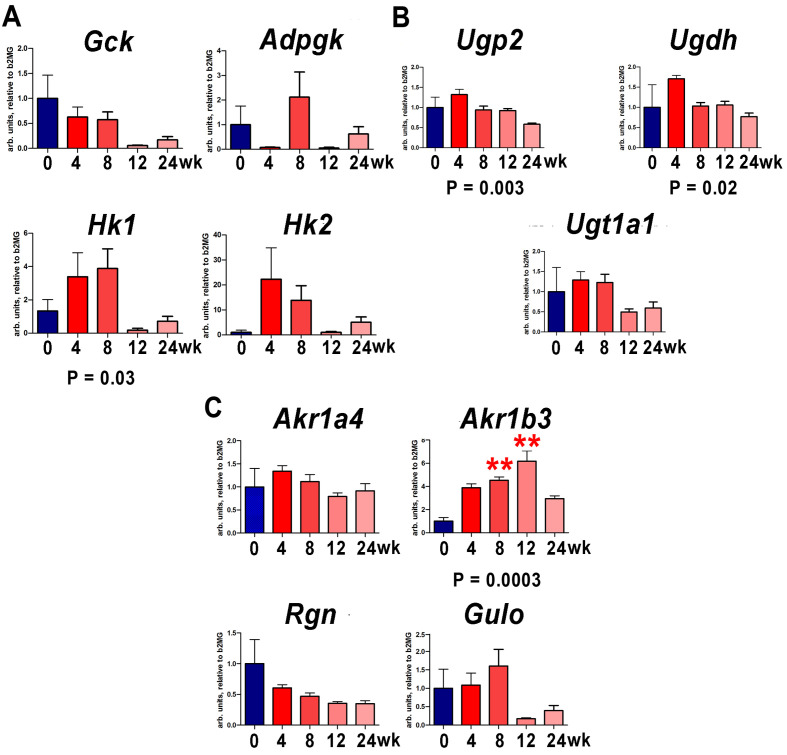
Expression of mouse genes involved in ascorbic acid synthesis from glucose and galactose after administration of 60% high-fat and choline-deficient amino-acid-defined diet. (**A**). The phosphorylation of hexoses pathway. (**B**). The synthesis of glucuronic acid from glucose 1-phosphate pathway. (**C**). The synthesis of ascorbic acid from glucuronic acid pathway. ** = *p* < 0.01 compared to controls. *p*-values represent nonparametric ANOVA (Kruskal–Wallis) analyses.

Figure 8 depicts the 24 week period in which hepatic gene expression was monitored during progressive fibrotic steatohepatitis due to feeding with a HF-CDAA diet. Of particular note is the four- and sixfold increase (*p* < 0.01) in *Akr1b3* (aldose reductase) expression by 8 and 12 week, respectively, a key enzyme in the synthesis of gulonic acid from glucuronic acid in the ascorbic acid synthesis pathway. Nonparametric ANOVA (Kruskal–Wallis test) showed an upward trajectory in *Akr1b3* expression throughout the administration of this diet (*p* = 0.0003). Smaller and less significant downward trends were observed in the expression of *Ugp2* (*p* = 0.003) and *Ugdh* (*p* = 0.02), two genes involved in the synthesis of glucuronic acid from glucose and galactose via glucose 1-phosphate (Figure 1). It would appear that significantly upregulated aldose reductase AKR1B3 expression mediates the increased synthesis of ascorbic acid from its hexose precursors.

### 3.4. Hepatic Gene Expression Changes with qRT-PCR after Withdrawal of a Choline-Deficient, Amino-Acid-Defined, 60% High-Fat Diet (HF-CDAA) and Spontaneous Reversal of F2 Fibrosis

**Figure 9:**
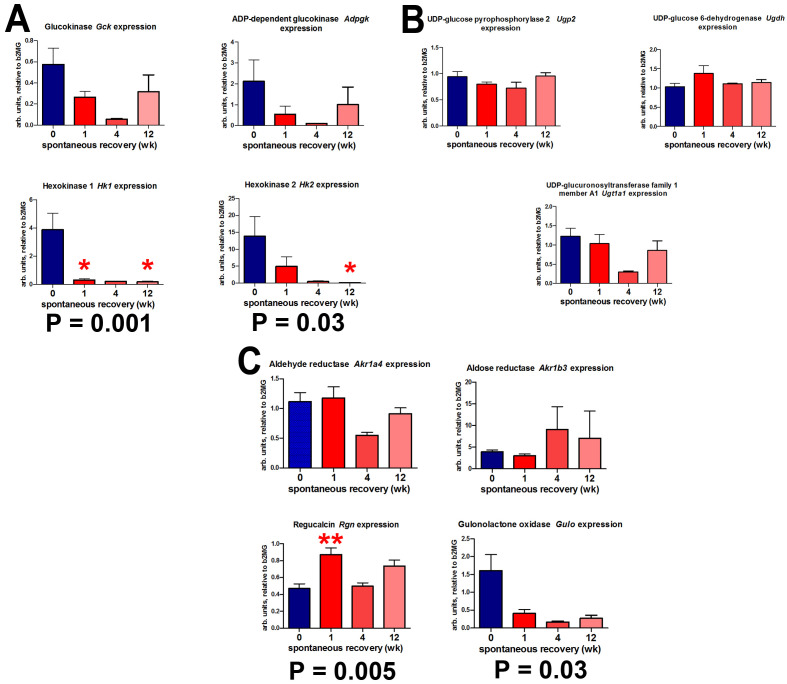
Expression of mouse genes involved in ascorbic acid synthesis from glucose and galactose during spontaneous recovery (for 1–12 week) from HF-CDAA diet-induced liver fibrosis. (**A**). The phosphorylation of hexoses pathway. (**B**). The synthesis of glucuronic acid from glucose 1-phosphate pathway. (**C**). The synthesis of ascorbic acid from glucuronic acid pathway. * = *p* < 0.05, ** = *p* < 0.01 compared to start of recovery phase (0 week). *p*-values represent nonparametric ANOVA (Kruskal–Wallis) analyses.

We have previously reported that mice with pre-established HF-CDAA-induced fibrosis undergo substantial tissue remodeling after switching to a normal diet, with a reversal of steatosis by week 4 and inflammatory cell infiltrates by week 12; fibrotic scar also underwent remodeling with splitting of fibrotic bands but persistence of collagen fibers in the liver up to 12 week. [16]. Figure 9 shows that expression of *Hk1* and *Hk2* fell dramatically within the 12 week recovery period, with an increase in *Rgn* expression in the same time period. By reversing the synthesis of gulonolactone from gulonic acid, regucalcin applies a brake on the synthesis of the further ascorbic acid (Figure 1) that is required for stabilizing the collagen triple helix by prolyl 4-hydroxylase. Although glucuronic acid continued to be synthesized in this period, the catabolism of ascorbic acid to threonic acid was clearly increased (Figure 5). Increased *Rgn* increased expression combined with elevated threonic acid production presumably limited ascorbic acid availability for prolyl 4-hydroxylase during the spontaneous recovery period limiting the formation of interstitial collagens previously reported during spontaneous recovery from F2 fibrosis [16].

## 4. Discussion

Here, we report that three mechanistically distinct liver disease models with progressive scarring in mice share a distinct metabolic reprogramming signature in the liver that was consistent with the diversion of glucose and galactose metabolism towards the synthesis of ascorbic acid across experimental liver fibrosis models, regardless of etiology. These metabolic changes promptly reversed upon the cessation of liver injury during the spontaneous recovery from advanced fibrosis, and in all four cases, were accompanied by specific changes in relevant metabolic gene expression.

Collagen represents the most common protein in animals and the modification of proline resides to yield (2*S*,4*R*)-4-hydroxyproline characterizes the single most prevalent post-translational modification in humans [33]. Prolyl 4-hydroxylase (procollagen-L-proline, 2-oxoglutarate: oxygen oxidoreductase; EC 1.14.11.2) is a Fe^2+^- and ascorbate-dependent dioxygenase that 4-hydroxylates proline residues in procollagen and stabilizes the collagen triple helix [13,33,37,38,39]. The mechanism of prolyl 4-hydroxylase and other ascorbate-related dioxygenases has been studied in great detail [40]. It is believed that the role of ascorbate in Fe^2+^-dependent dioxygenases is to reduce enzyme-bound Fe(III) to Fe(II) at the end of a reaction cycle, which permits the release of the active enzyme [41]. Contradictory findings in ascorbate-deficient *Gulo*^-/-^ mice that appear to continue to produce 4-hydroxyproline after vitamin C supplementation withdrawal have been reported in a single study [42]. These authors speculated that in the absence of ascorbate, alternative cellular reducing agents may be involved or that a second non-ascorbate-dependent prolyl hydroxylase may exist, although this has not been corroborated to date. However, extensive kinetic studies and other data are consistent with an ordered binding of Fe^2+^, 2-oxoglutarate, O_2_, and the peptide substrate to prolyl 4-hydroxylase, a stoichiometric consumption of ascorbate, followed by an ordered release of the reaction products [43].

The collagen content of liver increases four- to sevenfold in cirrhosis [44] associated with a three- to eightfold increased synthesis involving collagen prolyl 4-hydroxylase during predisposing conditions such as alcoholic liver disease and chronic hepatitis [45,46]. In the mouse model fed a HF-CDAA diet for 8 week, total hepatic collagen, determined by hydroxyproline content, rose 7.5-fold [16], and 3–4-fold when mice were administered escalating-dose CCl4 or TAA for 6 week [17]. Accordingly, the administration of TAA to mice twice weekly for 9 week resulted in a reported eightfold increase in hepatic procollagen I concentration [47]. Our metabolomic and gene expression findings are consistent with these reports of fibrogenesis related to CCl4 and TAA dosing, together with a HF-CDAA diet. For CCl4-induced fibrosis, the hepatic metabolic map showed decreases in the hexoses glucose, galactose and glucose, and a decrease in pentose phosphate pathway intermediates gluconic acid and ribose 5-phosphate. Although ascorbate was not detected in this experiment, its terminal metabolite threonic acid showed a clear increase. Collagen prolyl 4-hydroxylation converts 2-oxoglutarate to succinate, which was enhanced in this experiment, together with its downstream metabolites in the TCA cycle fumarate and malate. These findings are consistent with increased synthesis of ascorbate from glucose and galactose to drive collagen prolyl 4-hydroxylation in the underlying mechanism of CCl4-driven fibrogenesis. Of the 11 gene expressions determined for these pathways, CCl4 administration to mice statistically significantly increased hepatic aldose reductase *Akr1b3* expression, which, therefore, appeared to be a key regulatory step in increased supply of ascorbate for CCl4-induced fibrosis. Interestingly, it has been reported that the aldose reductase inhibitor (*Z*)2-(5-(4-methoxybenzylidene)-2,4-dioxothiazolidin-3-yl)acetic acid protected rats against CCl4-induced hepatic fibrosis. The authors, rather than focusing on the inhibition of aldose reductase in the ascorbate synthesis pathway from glucuronic acid, rationalized their experimental results differently, i.e., the modulation of NF-κB-dependent activation of inflammatory cytokines, an increase in hepatic glutathione and attenuation of oxidative stress [48]. In our study, TAA-induced fibrosis was associated with similar metabolic changes as CCl4 with diminished hepatic glucose, galactose, fructose, maltose (a disaccharide of glucose), the pentose phosphate pathway intermediates ribose 5-phosphate and xylose, and glucuronic acid, with elevated ascorbate. Again, as with CCl4, the TCA intermediates succinate, fumarate and malate were all increased. Because glucose was utilized in the synthesis of ascorbate, glycolysis was presumably downregulated and fatty acid oxidation was correspondingly upregulated with diminished hepatic concentrations of palmitic, linoleic and oleic acids. This was observed for both CCl4- and TAA-induced fibrosis. The observed changes in the metabolic pathway from glucose/galactose to ascorbate after TAA administration were supported by the finding of a fivefold increased expression of UDP-glucose 6-dehydrogenase *Ugdh*, involved in the synthesis of glucuronic acid, and an eightfold increased expression of aldose reductase *Akr1b3*, which apparently drained glucuronic acid levels in the synthesis of elevated ascorbate. Recently, it was reported that the silencing of glutathione *S*-transferase zeta (GSTZ1) using a CRISPR/Cas9-mediated *GSTZ1*-knockout in both human and mouse hepatoma cell lines, promoted cell migration in a mechanism involving UDP-glucose 6-dehydrogenase (UGDH)-mediated UDP-glucuronic acid accumulation [49]. Interestingly, our earlier metabolomic investigation of patients with chronic hepatitis C revealed a clear induction of the aldose reductase AKR1B10 with enhanced catabolism of both glucose and galactose [18], although the stage of hepatic fibrosis in these patients was not reported.

The administration of the HF-CDAA diet to mice over a period of 24 week provided the opportunity to examine metabolic and gene expression changes in relation to the entire natural history of chronic liver disease progression through fibrosis stages F1, F2, F3 and F4 (end-stage liver cirrhosis and liver cancer). During this advancing fibrosis, clear declining hepatic concentrations were found for glucose, galactose, fructose, galactose 1-phosphate, glucuronic acid, glucose 6-phosphate, glycolytic intermediates fructose 6-phosphate and glycerol 6-phosphate, together with the pentose phosphate pathway intermediate ribose 5-phosphate and the nucleoside adenosine. These metabolic enhancements resulted in increased ascorbate concentrations in the liver, supported by induced aldose reductase *Akr1b3* gene expression at 4 and 8 week. Therefore, the hijacking of the aforementioned energy molecules for the synthesis of ascorbate had knock-on effects on the levels of adenosine and the commonest long-chain fatty acids palmitic and oleic acid, which were reduced despite the feeding of a 60% high-fat diet. These two fatty acids are commonly used to generate an in vitro model of steatosis with HepG2 cells [50,51,52]. As with the CCl4 and TAA models, increased fatty acid oxidation has been upregulated to compensate for diminished glycolysis sequestered for the synthesis of ascorbate. This metabolic remodeling from carbohydrate to lipid oxidation en route to liver cirrhosis and HCC has been previously discussed [11].

A unified metabolomic model of liver fibrosis could be further tested by observing the metabolic changes that occurred when the fibrogenic provocation was removed. Cessation of the HF-CDAA diet at 8 week permitted observation of the metabolic and gene expression changes during spontaneous recovery from F2 fibrosis. Increases over 12 week in hepatic glucose, galactose, fructose, galactose 1-phosphate, galacturonic acid, glucose 6-phosphate, fructose 6-phosphate, ribulose 5-phosphate, glucuronic acid and threonic acid, together with a decline in ascorbic acid, all support the proposition that the HF-CDAA diet triggered hijacking of carbohydrate energy metabolism to support ascorbate production that was clearly and completely reversible on return of the mice to their normal chow. Moreover, *Hk1*, *Hk2* and *Gulo* expression were all diminished and the “brake” on ascorbate production, the gulonolactonase *Rgn*, was transcriptionally activated twofold, as early as 1 week after the diet switch.

Although mice are able to synthesize ascorbate, scorbutic mice were generated by *Gulo* gene inactivation (*Gulo*^-/-^) and ascorbate dietary restriction. Such mice had compromised collagen synthesis and, therefore, extracellular matrix production. Xenotransplanted mouse melanoma B16FO and mouse breast cancer 4T1 cells were both highly metastatic in host scorbutic female Balb/C mice but dietary supplementation with ascorbic acid, sufficient to raise serum concentrations 50-fold, reduced metastasis of the melanoma cells by 71% and breast cancer cells by 28% [53]. As with collagen prolyl 4-hydroxylase, ascorbate is also an obligate cofactor for other proline hydroxylase domain (PHD) proline hydroxylases in the Fe(II) and 2-oxoglutarate-dependent oxygenase superfamily, such as the HIF proline hydroxylases [54]. It was previously reported that HF-CDAA diet administration to C57BL/6J male mice generated hepatocellular carcinomas at 24 week in 80% of the mice [16]. The question remains: what role, if any, did enhanced ascorbate production play in the generation of these tumors on the cirrhotic background at 24 week when maximum metabolic remodeling had occurred from hexose metabolism to ascorbate synthesis? The recent report of a low-molecular-weight inhibitor 34-132D of gene transcription by HIF-1 and HIF-2 in mouse HCC tumors and its role in tumor eradication [55] demonstrated a facet of the relationship between HIF and HCC. HIF-1α is a transcription factor essential for cancer cell survival [56]. Prolyl 4-hydroxylation is necessary not only for proper structural assembly of collagens but also for oxygen-dependent protein stability of transcription factors such as HIF-1α. It is, therefore, likely that enhanced flux to ascorbate in mouse liver promoted HIF-1α levels by contributing to PHD enzyme activity. However, it has been reported that *Gulo*^-/-^ mice have normal HIF-dependent patterns of gene expression similar to ascorbate-supplemented mice. Furthermore, ascorbate could be substituted by glutathione in vitro for all three isoforms of PHD [57] but neither glutathione nor dithiothreitol displayed the efficacy of ascorbate in this regard [58]. Other investigators have shown that physiological concentrations of ascorbate are sufficient to increase the activity of PHD enzymes in downregulating HIF-1α [59]. Finally, in renal cell carcinoma cells, it was reported that the hypoxic pathway can be regulated by increasing HIF hydroxylase activity via intracellular ascorbate availability [60].

The involvement of tryptophan metabolism in liver fibrosis has been reported on several occasions [61], both in CCl4-treated rats [62] and in patients with cirrhosis [63]. It is of interest to note that the rate-limiting and initial step in the kynurenine pathway of tryptophan catabolism is mediated principally by two 2,3-dioxygenases, tryptophan 2,3-dioxygenase and indoleamine 2,3-dioxygenase. These are both heme-containing enzymes that require ascorbate to reduce inactive Fe(III) to the active Fe(II) [64]. This is in parallel with the aforementioned role of ascorbate as a reducing agent for collagen prolyl 4-hydroxylase. It could be speculated that tryptophan catabolism is associated with liver fibrosis in both patients and experimental rodent models because of metabolic switching from glucose and galactose energy metabolism to ascorbate production, as demonstrated here.

Increased metabolic flux from glucose and galactose to ascorbate in three independent experimental mouse models drove the observed fibrogenesis in the liver, as determined by both metabolomic and gene expression outcomes. When disease in the F2 fibrotic liver was permitted to spontaneously reverse, both metabolomic and gene expression findings mirrored the findings in progressive fibrogenesis experiments. The shunting of hexose metabolism towards ascorbate production in the mouse liver appears to be an important contributor to liver scarring and unifying metabolic signature of fibrogenesis. Further investigations would be required to understand if this metabolic hijacking of hexose metabolism contributes to hepatocellular carcinoma formation.

This work has cast a new light on the mechanisms of fibrogenesis in the mouse in relation to the metabolic supply of ascorbate. However, humans, together with primates, certain bats, and the guinea pig, are alone among mammals in lacking L-gulono-1,4-lactone oxidase activity, rendering them unable to synthesize ascorbate [65,66]. Humans, therefore, depend upon dietary sources of ascorbate to fulfill its many biochemical, epigenetic and physiological roles. Deficiency in dietary ascorbate leads to scurvy, a condition with signs and symptoms related to impaired collagen production. Despite the dichotomy in ascorbate availability between man and the rodent models for human liver disease, our findings nevertheless may have relevance to the progression of liver fibrosis and development of hepatocellular carcinoma in man. A genome-wide meta-analysis identified 11 single-nucleotide polymorphisms (SNPs) associated with plasma vitamin C concentration, which were used to investigate the risk of type 2 diabetes [67], cardioembolic stroke and Alzheimer’s disease [68]. In the case of type 2 diabetes, of the 11 genomic regions association with ascorbate plasma concentration in 80,983 cases and 842,909 noncases, the strongest signal was found with *SLC23A1* [67], the vitamin C transporter SVCT1, with a significant signal also with *SLC23A3*, the orphan transporter SVCT3 [69]. Plasma ascorbate was inversely associated with type 2 diabetes [67]. The SNPs in *SLC23A1* (rs33972313) and *SLC23A3* (rs13028225) are polymorphic with C>A,G,T [70] and T>C alleles [71], respectively. If these genotypes are expressed as functional phenotypes, the delivery of ascorbate from the diet to the liver might be polymorphic and influence the progression of fibrotic liver disease, as we observed in the mouse. A genome-wide analysis of liver fibrosis in relation to ascorbate plasma concentration is yet to be conducted. In contrast, three genome-wide association studies for cirrhosis have been reported [72,73,74]. In one study, 12 genetic variants were associated with cirrhosis and were used to identify those most at risk from the hepatotoxic effects of excess alcohol consumption or obesity [72]. A single genome-wide association study on progression of liver fibrosis from HCV infection reported seven genetic variants that the authors stated were involved in the regulation of apoptosis, concluding that control of apoptosis might be involved in liver fibrosis [75].

As we have demonstrated here, hepatic ascorbate concentrations in the mouse depend largely on de novo synthesis from glucose and related hexoses. We report here for the first time that ongoing chronic liver injury from CCl4, TAA or a HF-CDAA diet increases the flux in this pathway to satisfy the requirements for intense de novo collagen synthesis during the progressive fibrotic response to these injuries. This is accomplished by the induction of both UDP-glucose 6-dehydrogenase and aldose reductase. After withdrawal of a fibrogenic stimulus, recovery involved immediate downregulation of hexokinase 1 and 2 expression together with upregulated transcription of regucalcin, which has gulonolactonase activity and, therefore, puts a brake on further ascorbate synthesis. Interestingly, in the guinea pig, which, like humans, cannot synthesize ascorbate, the administration of a very-high-sucrose diet did not provoke stage F1 to F3 fibrosis, in contrast to various high-fat diets [76]. In stark contrast, feeding a high-sucrose diet to mice resulted in increased COL1A1 and mild fibrosis production compared to mice on a control diet or ketohexokinase gene (*Khk*) knockout mice fed the high-sucrose diet [77]. This species difference underlines the importance of ascorbate in the genesis of liver fibrosis. In humans, there is evidence that the delivery to the liver of ascorbate by SLC23A transporters accompanies chronic liver disease [78]. As has already been accomplished for type 2 diabetes, stroke and Alzheimer’s disease, an investigation of the association of plasma ascorbate concentration and liver disease is warranted.

## Figures and Tables

**Table 1:** Hepatic metabolite levels on a normal diet only (ND) and after administration of carbon tetrachloride (CCl4).

Metabolite	ND Mean ± Sem	CCl4 Mean ± Sem	Fold- Change	*p*-Value (Uncorrected)
Glucose	137.8 ± 13.57	104.0 ± 3.80	−1.3	0.03
Glucose 6-phosphate	0.41 ± 0.09	0.15 ± 0.03	−2.7	0.01
Fructose	4.9 ± 0.92	1.1 ± 0.05	−4.5	0.001
Gluconic acid	2.6 ± 0.30	0.93 ± 0.10	−2.8	0.0001
*myo*-Inositol	1.1 ± 0.17	1.9 ± 0.16	+1.7	0.006
Galactose	42.54 ± 3.00	30.06 ± 1.42	−1.4	0.002
Galactose 1-phosphate	0.71 ± 0.10	0.35 ± 0.06	−2.0	0.007
Galacturonic acid	1.3 ± 0.19	0.32 ± 0.02	−4.1	0.0001
Maltose	1.7 ± 0.58	0.29 ± 0.03	−5.9	0.03
Ribose 5-phosphate	1.3 ± 0.17	0.45 ± 0.03	−2.9	0.0002
Ribitol	0.22 ± 0.02	0.14 ± 0.01	−1.6	0.02
Glycine	20.84 ± 1.53	11.87 ± 0.90	−1.8	0.0002
Glutamic acid	4.6 ± 0.67	8.8 ± 1.3	+1.9	0.01
Palmitic acid	1.0 ± 0.11	0.55 ± 0.04	−1.8	0.002
Linoleic acid	0.13 ± 0.02	0.04 ± 0.01	−3.3	0.0001
Succinic acid	5.8 ± 0.62	14 ± 1.5	+2.4	0.0003
Fumaric acid	1.5 ± 0.12	2.6 ± 0.23	+1.7	0.0007
Malic acid	4.5 ± 0.37	7.5 ± 1.0	+1.6	0.02
2-Hydroxyglutaric acid	0.15 ± 0.01	0.39 ± 0.03	+2.6	<0.0001
Glycerol 3-phosphate	7.8 ± 0.66	12 ± 1.0	+1.5	0.01
2-Aminobutanoic acid	0.79 ± 0.08	0.38 ± 0.07	−2.1	0.002
Threonic acid	1.80 ± 0.16	0.74 ± 0.05	−2.4	<0.0001
Uracil	0.30 ± 0.03	0.55 ± 0.03	+1.8	<0.0001

**Table 2:** Hepatic metabolite levels on a normal diet only (ND) and after administration of thioacetamide (TAA).

Metabolite	ND Mean ± Sem	TAA Mean ± Sem	Fold Change	*p*-Value (Uncorrected)
Glucose	138 ± 14	95 ± 3.4	−1.5	0.0003
Fructose	4.9 ± 0.92	1.2 ± 0.11	−4.1	<0.0001
Glucuronic acid	1.9 ± 0.19	1.1 ± 0.06	−1.7	<0.0001
Ascorbic acid	4.2 ± 0.44	6.8 ± 0.35	+1.6	0.0002
Galactose	43 ± 3.0	28 ± 1.0	−1.5	<0.0001
Galacturonic acid	1.3 ± 0.19	0.54 ± 0.05	−2.4	<0.0001
Maltose	1.7 ± 0.58	0.26 ± 0.02	−2.9	0.001
Ribose 5-phosphate	1.3 ± 0.17	0.51 ± 0.02	−2.5	<0.0001
Xylose	0.55 ± 0.07	0.19 ± 0.01	−2.9	<0.0001
Glutamic acid	4.6 ± 0.67	13 ± 1.3	+2.8	0.0002
Proline	3.6 ± 0.38	6.2 ± 0.46	+1.7	0.002
Palmitic acid	1.0 ± 0.11	0.58 ± 0.02	−1.7	<0.0001
Oleic acid	0.20 ± 0.03	0.12 ± 0.01	−1.7	0.0001
Linoleic acid	0.13 ± 0.02	0.05 ± 0.01	−2.6	<0.0001
Succinic acid	5.8 ± 0.62	13 ± 0.72	+2.3	<0.0001
Fumaric acid	1.5 ± 0.12	2.3 ± 0.15	+1.5	0.003
Malic acid	4.5 ± 0.37	5.9 ± 0.31	+1.3	0.02
2-Hydroxyglutaric acid	0.15 ± 0.01	0.60 ± 0.05	+4.0	<0.0001
Uracil	0.30 ± 0.03	0.72 ± 0.05	+2.4	<0.0001
Uridine	0.67 ± 0.13	1.5 ± 0.12	+2.2	0.0003

**Table 3:** Hepatic metabolite changes in a HF-CDAA model of fibrosis.

Metabolite	ND	F1	F2	F3	F4	Kruskal–Wallis One-Way ANOVA *p*-Value
	Mean ± Sem	Mean ± Sem	Mean ± Sem	Mean ± Sem	Mean ± Sem	
Glucose	138 ± 14	111 ± 11	59 ± 6.0	73 ± 8.3	75 ± 5.0	<0.0001
Fructose	4.9 ± 0.92	1.4 ± 0.26	0.59 ± 0.1	0.63 ± 0.1	0.68 ± 0.10	<0.0001
Glucuronic acid	1.9 ± 0.19	0.95 ± 0.04	0.75 ± 0.07	0.60 ± 0.06	0.75 ± 0.04	<0.0001
Ascorbic acid	4.2 ± 0.44	10 ± 0.35	8.3 ± 0.53	9.6 ± 0.63	8.4 ± 0.53	0.0004
Galactose	43 ± 3.0	30 ± 2.0	18 ± 1.6	21 ± 1.5	25 ± 1.50	<0.0001
Glucose 6-phosphate	0.41 ± 0.10	0.16 ± 0.02	0.07 ± 0.01	0.08 ± 0.03	0.09 ± 0.01	<0.0001
Fructose 6-phosphate	0.93 ± 0.12	0.60 ± 0.09	0.23 ± 0.04	0.27 ± 0.07	0.30 ± 0.03	<0.0001
Galactose 1-phosphate	0.71 ± 0.10	0.42 ± 0.04	0.17 ± 0.03	0.20 ± 0.06	0.19 ± 0.03	<0.0001
Ribose 5-phosphate	1.3 ± 0.17	0.85 ± 0.10	0.38 ± 0.05	0.27 ± 0.04	0.26 ± 0.03	<0.0001
Aspartic acid	0.39 ± 0.04	0.93 ± 0.1	0.96 ± 0.06	0.97 ± 0.1	0.79 ± 0.05	0.001
Palmitic acid	1.0 ± 0.11	0.61 ± 0.05	0.59 ± 0.07	0.42 ± 0.05	0.27 ± 0.02	<0.0001
Oleic acid	0.20 ± 0.03	0.15 ± 0.01	0.17 ± 0.02	0.09 ± 0.01	0.08 ± 0.01	0.002
Citric acid	1.2 ± 0.13	0.83 ± 0.04	0.48 ± 0.05	0.36 ± 0.04	0.36 ± 0.04	<0.0001
Glycerol 3-phosphate	7.8 ± 0.66	5.0 ± 0.40	2.4 ± 0.25	3.5 ± 0.57	3.5 ± 0.37	<0.0001
Creatinine	0.23 ± 0.04	0.54 ± 0.06	0.57 ± 0.03	0.48 ± 0.04	0.49 ± 0.03	0.0002
Uracil	0.30 ± 0.03	0.56 ± 0.03	0.46 ± 0.03	0.41 ± 0.03	0.34 ± 0.04	0.0006
Adenosine	0.35 ± 0.06	0.20 ± 0.02	0.06 ± 0.01	0.07 ± 0.02	0.10 ± 0.02	<0.0001

ND means normal diet.

## Data Availability

Data can be made available by request to the corresponding author.

## Supplementary Materials

The following supporting information can be downloaded at: https://www.mdpi.com/article/10.3390/cells12030485/s1, Supplementary Table S1: Details of assays and primers for qRT-PCR analysis of mouse livers.
